# Activatable cell–biomaterial interfacing with photo-caged peptides†
†Electronic supplementary information (ESI) available. See DOI: 10.1039/c8sc04725a


**DOI:** 10.1039/c8sc04725a

**Published:** 2018-11-16

**Authors:** Yiyang Lin, Manuel M. Mazo, Stacey C. Skaalure, Michael R. Thomas, Simon R. Schultz, Molly M. Stevens

**Affiliations:** a Department of Materials , Department of Bioengineering and Institute for Biomedical Engineering , Imperial College London , Exhibition Road , London SW7 2AZ , UK . Email: m.stevens@imperial.ac.uk; b Department of Bioengineering and Centre for Neurotechnology , Imperial College London , London SW7 2AZ , UK

## Abstract

Spatio-temporally tailoring cell–material interactions is essential for developing smart delivery systems and intelligent biointerfaces. Here we report new photo-activatable cell–material interfacing systems that trigger cellular uptake of various cargoes and cell adhesion towards surfaces. To achieve this, we designed a novel photo-caged peptide which undergoes a structural transition from an antifouling ligand to a cell-penetrating peptide upon photo-irradiation. When the peptide is conjugated to ligands of interest, we demonstrate the photo-activated cellular uptake of a wide range of cargoes, including small fluorophores, proteins, inorganic (*e.g.*, quantum dots and gold nanostars) and organic nanomaterials (*e.g.*, polymeric particles), and liposomes. Using this system, we can remotely regulate drug administration into cancer cells by functionalizing camptothecin-loaded polymeric nanoparticles with our synthetic peptide ligands. Furthermore, we show light-controlled cell adhesion on a peptide-modified surface and 3D spatiotemporal control over cellular uptake of nanoparticles using two-photon excitation. We anticipate that the innovative approach proposed in this work will help to establish new stimuli-responsive delivery systems and biomaterials.

## Abstract 2

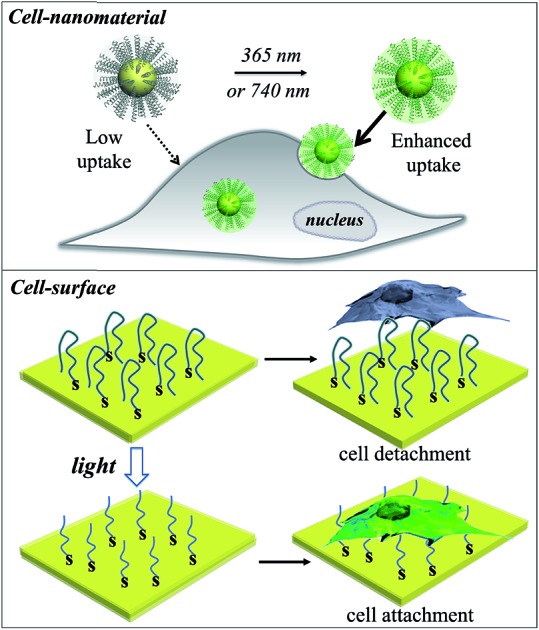
We report an effective strategy to design activatable cell–material interfacing systems enabling photo-modulated cellular entry of cargoes and cell adhesion towards surfaces.

## Introduction

The recent explosive growth of research in the field of nanotechnology has provided a wide range of novel materials and strategies for biomedicine, including important advances in bioimaging, drug delivery, photothermal/photodynamic therapy, and gene transfection.^1^–^7^ However, further translation beyond basic research is heavily hampered by the inefficient performance of nanomaterials in biological environments. Amongst the main hurdles, the hydrophobic nature of the lipid bilayer of the plasma membrane renders it impermeable to most polar, hydrophilic molecules including peptides, proteins, oligonucleotides, drugs and nanomaterials that lack specific membrane receptors or transport mechanisms. Cell-penetrating peptides (CPPs) such as the HIV transactivator of transcription (TAT) peptide and arginine oligomer protein-transduction domain have been widely used as transport vector tools for the cellular import of a variety of cargos (*e.g.*, nanomaterials and biomolecules) through the cell membrane.^8^,^9^ In addition, new cell-penetrating transporters have been developed such as synthetic peptides,^10^,^11^ helical poly(arginine) mimics,^12^ antibiotics,^13^ poly-(disulfide)s^14^,^15^ and guanidinium-containing synthetic polymers.^16^,^17^ In spite of their potential, the inherent non-specificity of CPPs has restricted their application in targeted delivery systems. An alternative strategy to selectively enhance cell–material interfacing lies in the design of smart systems that are triggered by external stimuli, or specific features of the target cell or local tissue physiology. For example, the development of acid-activated CPPs takes advantage of the acidic tumour extracellular environment^18^,^19^ for tumour-targeted drug delivery.^20^–^22^ Among these, pH-induced charge reversal and pH-sensitive transmembrane insertion of a low-pH insertion peptide have also been explored as novel strategies to increase the efficacy of biomaterial administration.^19^ Similarly, the design of hydrogen peroxide (H_2_O_2_) and matrix metalloproteinase-2 (MMP2) responsive cellular delivery systems have been reported.^23^,^24^ Key attributes of such smart delivery systems are their ability to control cellular entry of biomolecules/nanomaterials and their release on demand, minimize side effects and improve the therapeutic efficacy of pharmaceuticals.

**Scheme 1:**
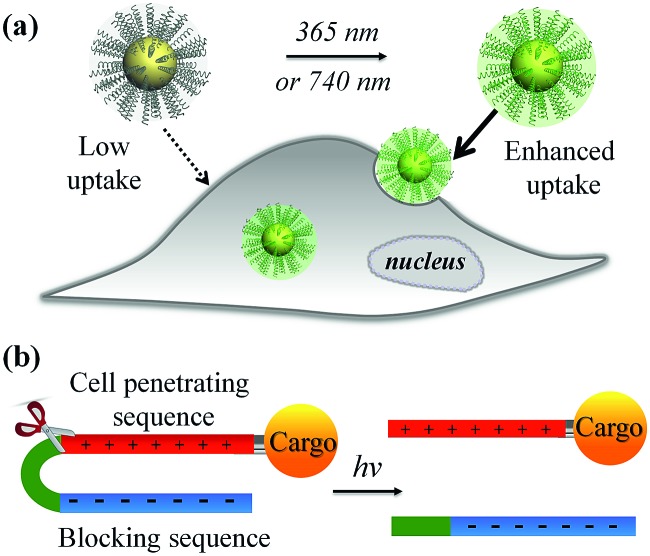
(a) Schematic of light-activated cellular uptake of biomolecules and nanomaterials. (b) Light-responsive delivery systems are functionalised with a synthetic ligand consisting of a negative blocking sequence, a light-cleavable sequence, and a cell penetrating peptide (CPP).

In this work, we developed a stimuli-responsive cell–material interfacing system enabling the spatial and temporal control of cellular delivery and cell attachment *via* photo-activation. Photo-stimulation of biomaterials is advantageous since it can be manipulated precisely, enabling fine control over irradiated volumes.^25^–^33^ In the presence of photo-stimulation, it is possible to rapidly increase the concentration of the active form of molecules with strict control over the illuminated area, time, and dosage.^34^–^38^ In our system, we synthesized a series of photo-caged peptide ligands consisting of a cell penetrating sequence, a blocking sequence, and a photo-cleavable linker (Scheme 1), and conjugated them to various nanomaterials or surfaces. The cell–material interaction is effectively suppressed when there is no light activation, resulting in minimal cargo uptake. Upon photo-irradiation, the linker is cleaved to remove the blocking peptide, resulting in activation of the cell penetrating peptide and increased cellular uptake. This phenomenon was demonstrated with a wide range of cargos including small and large molecules, organic and inorganic nanoparticles, and self-assembled soft materials.

## Results and discussion

**Fig. 1:**
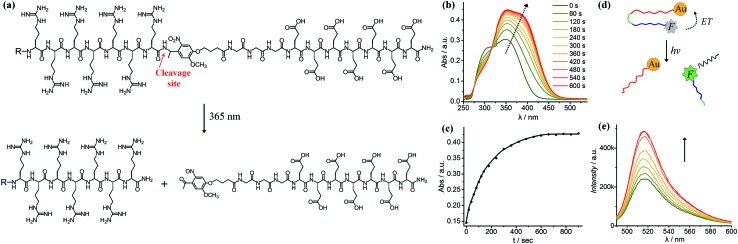
(a) Structure of the light-cleavable ligand, where the blocking peptide contains hepta-glutamic acid (E7) and the CPP contains hepta-arginine (R7). The photo-cleavable spacer contains an *o*-nitrobenzyl group and a triglycine. The photo-cleavage site is indicated by an arrow. After light irradiation, the glutamic acid-rich sequence is removed to generate a highly positive R7 with a C-terminal amidation. (b) UV-vis spectra showing the kinetics of ligand cleavage under 365 nm light over a period of 10 min. (c) The increase of UV-vis absorbance at 390 nm indicates that the photo-triggered reaction is completed within 10 min. (d and e) The fluorescence recovery test shows that the removal of the blocking sequence from AuNPs after light irradiation (*t* = 0, 1, 3, 6, 10, 20, 25 and 30 min) increases fluorescence.

As shown in Fig. 1a, we synthesised a photo-labile ligand consisting of hepta-arginine (R7), hepta-glutamic acid (E) and a photo-linker. The positive R7 serves as the cell penetrating sequence capable of delivering cargos to cells either by direct membrane translocation or promoting endocytosis.^39^ The proximal glutamic acid-rich sequence (E7) blocks the oligoarginine by electrostatic attraction, forming a U-shaped antifouling zwitterionic ligand.^40^ The cell penetrating peptide (R7) and the blocking sequence (E7) are connected with a photo-cleavable linker, which can be cleaved at the position of the *o*-nitrophenyl group to yield two peptide chains terminated with ketone and amide groups (Fig. 1a). We monitored the photo-cleavage reaction with UV-vis spectroscopy over a period of 15 min (Fig. 1b). Without UV light irradiation, there are two absorption peaks at approximately 305 nm and 350 nm. Upon UV irradiation, the absorbance at 305 nm decreased and the peak intensity at 350 nm increased, while a shoulder peak emerged at 370–400 nm. Since arginine and glutamic acid do not have an absorption peak between 300 nm and 400 nm, these spectral changes originate from the photo-cleavage of the *o*-nitrophenyl group. The isosbestic point at 317 nm indicates no side reactions or decomposition in the process of photo-irradiation. The reaction reached a photostationary state after 10 min, as the absorbance at 390 nm approached a plateau (Fig. 1c). Because the electrostatic interactions between hepta-glutamic acid (E7) and hepta-arginine (R7) are strong due to multiple charge pairs, it is important to determine whether the negative blocking peptide will electrostatically bind to the cell penetrating peptide even when the covalent spacer is cleaved. To test this phenomenon, we developed a FRET system using a fluorescein isothiocyanate-labelled, thiolated peptide (HS-R7E7-FITC) and citrate-coated gold nanoparticles (∼40 nm) (Fig. S1† and 1d). The FITC-appended peptide was covalently bound to AuNPs *via* the Au–thiol bond, and the close proximity between FITC and AuNPs enabled efficient energy transfer (ET) from FITC to AuNPs, resulting in quenched FITC fluorescence. Upon exposure to UV light, FITC fluorescence increased with irradiation time (Fig. 1e), suggesting that the FITC-conjugated blocking peptide (E7) was released from the hepta-arginine sequence following cleavage of the nitrophenyl group.

**Fig. 2:**
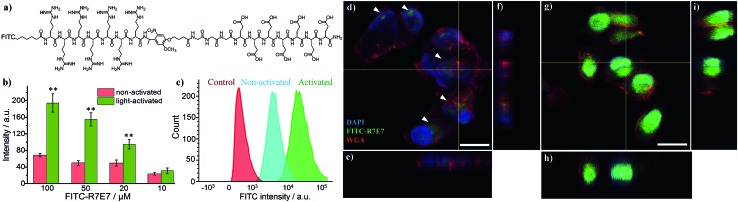
(a) Structure of the FITC-labelled photo-cleavable peptide (FITC-R7E7). (b) Cellular uptake of FITC-R7E7 by MDA-MB-231 cells at different concentrations (*i.e.*, 100, 50, 20 and 10 μM) before and after light activation for 10 min was evaluated by quantifying cellular fluorescence intensity. (c) FACS data and (d–i) confocal images of MDA-MB-231 cells highlight the distinct difference in FITC internalization when cells were incubated with 50 μM FITC-R7E7 in the absence of (d–f) and following (g–i) light irradiation. Scale bar: 20 μm. **: *p* < 0.01 non-activated *vs.* activated. The nucleus is stained with DAPI (4′,6-diamidino-2-phenylindole, dihydrochloride); the plasma membrane is stained with the probe WGA (wheat germ agglutinin).

Since the blocking peptide could be removed with 365 nm UV light, we anticipated that the bioactivity of CPP (R7) could be enhanced through controlled light irradiation. To test this idea, we synthesised a fluorescein isothiocyanate-labelled photo-activatable peptide (FITC-R7E7) with FITC attached to the N-terminus (Fig. S1† and 2a). Peptide solutions at different concentrations (100, 50, 20 and 10 μM) were either exposed to light or left unexposed and incubated with MDA-MB-231 (MDA) cells. After 24 h, we evaluated the level of internalized fluorophores by measuring the fluorescence intensity of cell samples. We found that photo-irradiation enhanced the cellular uptake by 1.1 to 3-fold for FITC-R7E7 concentrations between 10 and 100 μM, respectively (Fig. 2b). Fluorescence activated cell sorting (FACS) confirmed that cells incubated with the activated peptide showed a higher fluorescence intensity than those incubated with the native construct (Fig. 2c). In addition, confocal microscopy showed the subcellular localization of the fluorophore, by staining the cell membrane with WGA (wheat germ agglutinin) and the nucleus with DAPI (4′,6-diamidino-2-phenylindole, dihydrochloride). MDA cells incubated with the non-activated peptides exhibited a weak and scattered green fluorescence signal (Fig. 2d–f, arrowheads), whereas cells incubated with light-activated FITC-R7E7 (Fig. 2g–i) exhibited high penetration of the fluorophore, with a preferential perinuclear localization (co-localization with DAPI; see orthogonal views in Fig. 2h and i). In a control experiment, we demonstrated that light irradiation did not cause any obvious fluorescence changes in the FITC-labelled peptide (Fig. S2†). Similarly, we demonstrated that light irradiation enhanced cellular internalization of the fluorophore with HeLa cells (Fig. S3†). These results show that although neutral peptide-bound FITC can be taken up by the cells to some degree, light-triggered cleavage of the blocking sequence significantly increases the quantity that translocates into MDA cells.

We further applied this strategy to control the intracellular delivery of large biomolecules. We used avidin, a di/tetrameric biotin-binding protein, 66–69 kDa in size, as a model macromolecule to test the possibility of light-activated protein delivery. To this end, we conjugated FITC-labelled avidin with 4 equivalents of the biotinylated photo-labile ligand (biotin-R7E7) *via* biotin–avidin affinity and incubated with MDA cells (Fig. S1†). After 24 h, cell fluorescence increased by up to 200% for FITC–avidin concentrations of 2.5 and 1.25 μM (Fig. S4†), highlighting the feasibility of this strategy to deliver full-length proteins.

**Fig. 3:**
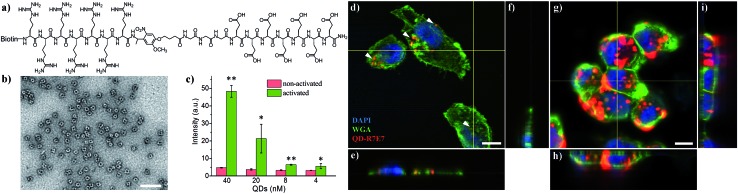
(a) Molecular structure of the biotinylated photo-cleavable ligand containing hepta-arginine, hepta-glutamic acid, and a photo-responsive linker (biotin-R7E7). (b) TEM image (negative staining) showing the core-corona type streptavidin-coated QDs. (c) Fluorescence of cells after internalizing photo-cleavable ligand modified QDs. (d–i) Confocal images of MDA cells showing the distinct internalization of QDs without (d–f) and following (g–i) light irradiation. Scale bar: 100 nm in (b) and 10 μm in (d–i). *: *p* < 0.05; **: *p* < 0.01 non-activated *vs.* activated.

We subsequently applied the light-activated peptide to control intracellular entry of inorganic nanoparticles. As an example, we coupled the cleavable peptide ligand to semiconducting CdSe/ZnS QDs. Compared to organic fluorophores, inorganic QDs possess a number of superior properties such as bright emission, high stability, reduced photobleaching and ease of surface functionalization.^41^ For example, the biotinylated photo-cleavable ligand was attached to streptavidin-coated QDs (5 nm in diameter, *λ*_em_ = 605 nm) *via* streptavidin–biotin affinity interactions (Fig. 3a and b). As shown in Fig. 3c, light irradiation increased the cellular uptake of QDs to 1015% and 573% when using 40 and 20 nM QDs, respectively. This increase in fluorescence is not attributed to enhanced QD emission after light irradiation (Fig. S5†). It is noteworthy that the light-triggered increase in QD uptake was strikingly higher than that of FITC–avidin as shown in Fig. S4,† which is ascribed to the high number of biotin-binding sites on QDs. According to the vendor, there are 5–10 covalently bound streptavidins per QD, permitting the attachment of 20–40 biotin-R7E7 molecules on a single QD. QD uptake by MDA cells was examined with confocal microscopy (Fig. 3d–i), where non-activated QDs were poorly taken up (Fig. 3d–f, arrowheads), and strong uptake was observed for cells incubated with the light-activated QD-peptide (Fig. 3g–i). To confirm the functionality of our system in a biologically relevant environment, we incubated the QD-R7E7 complexes with MDA cells and irradiated them *in situ*. The results were consistent with our previous observations, where FACS indicated a strong fluorescence signal per cell (Fig. S6†), indicating increased uptake of the activated QDs. There was however a notable difference in the uptake pattern between activated QD/biotin-R7E7 and activated FITC-R7E7, where cells showed a punctate and mainly cytosolic distribution of QDs in contrast with the preferentially nuclear distribution of FITC. The reasons behind this are unclear, but it is reasonable to hypothesize that differences in the cargo size and CPP concentration could affect whether the QDs are primarily taken up *via* endocytosis or direct membrane translocation.^39^

Our light-cleavable ligands have broad applicability for improving intracellular uptake of a variety of nanomaterials, which can be bound to the peptide using well-established conjugation chemistry (Table 1). For example, carboxylated polystyrene particles modified with an aminated ligand (H_2_N-R7E7, Fig. S1†) displayed enhanced cellular uptake after light activation (Fig. S7†). Branched Au nanostars with a localised surface plasmon resonance peak at 820 nm in the infrared range (so-called “biological window”) were internalized with a greater efficiency following light irradiation (Fig. S8†). The Au nanostars were conjugated *via* thiolated ligands (HS-R7E7). Similarly, we demonstrated light-enhanced delivery of lipid vesicles (∼200 nm size) by conjugating HS-R7E7 *via* the thiol–maleimide reaction (Fig. S9†). In all cases, light-triggered cleavage of the nitrophenyl group and subsequent removal of the blocking peptide greatly promoted the cellular uptake of nanomaterials.

**Table 1:** Photo-activated cellular uptake was demonstrated for a wide range of biomolecules and nanomaterials with different sizes (*d*). The concentrations of FITC, avidin, QDs, PS, Au stars and liposomes were 100 μM, 2.5 μM, 40 nM, 50 μg mL^–1^, 2.85 nM and 0.5 mg mL^–1^. The enhancement rate (*η*) was determined by comparing the cellular uptake with and without light activation

	FITC	Avidin	QDs	PS	Au stars	Liposomes
*d* (nm)	1	5	10	20	50	200
*η* (%)	283	207	1015	1063	297	290

**Fig. 4:**
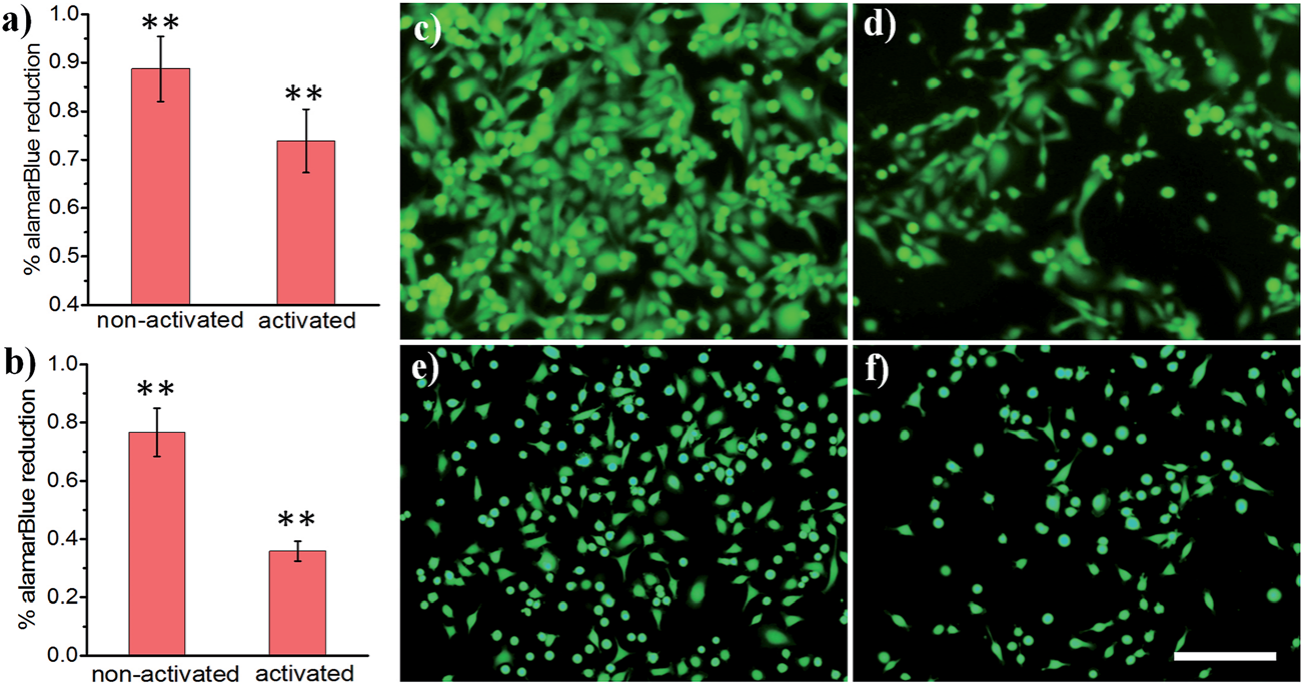
(a and b) alamarBlue® reduction of cancer cells treated with ligand-coated PLGA particles: (a) MDA and (b) HeLa. (c–f) Representative images of calcein AM-loaded cancer cells treated with drug-loaded PLGA nanoparticles: (c and d) MDA and (e and f) HeLa cells; (c and e) non-activated and (d and f) light-activated. Scale bar: 200 μm. **: *p* < 0.01 non-activated *vs.* activated.

Light-activated cellular uptake strategies enable targeted delivery of nanomaterials to diseased tissue, which could minimize off-target side effects and also potentially decrease the dosage administered to a patient. In this work, we prepared phospholipid-coated polylactic-*co*-glycolic acid (PLGA) nanoparticles as a drug carrier for camptothecin, a cytotoxic alkaloid employed in cancer therapy. However, this drug has poor water solubility which limits its efficacy.^42^ PLGA particles are attractive polymer-based delivery systems because of their biocompatibility, efficient encapsulation of hydrophobic materials, and tunable drug release properties. We synthesized PLGA particles *via* emulsion/solvent evaporation methods, using lecithin and DSPE-PEG-NHS as surfactants. We further conjugated a light-activatable peptide ligand to PLGA particles by reacting an aminated peptide (H_2_N-R7E7) with DSPE-PEG-NHS. As shown in Fig. 4a, c and d, exposing drug-loaded polymer particles to 365 nm UV light resulted in enhanced MDA cell death, as demonstrated by the statistically significant decrease in alamarBlue® reduction activity. A similar trend was observed for HeLa cells (Fig. 4b, e and f), and it is likely that the difference in the magnitude of effect was determined from differential entry propensities and drug sensitivities between the two cell lines.

**Fig. 5:**
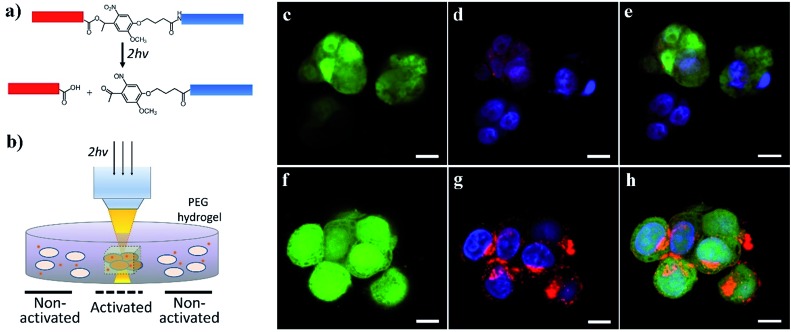
Two-photon photoactivation experiments: (a) cleavage of the *o*-nitrobenzyl ether bond by focused two-photon excitation (740 nm), which removes the blocking peptide (blue) from the cell-penetrating peptide (red). (b) GFP-HeLa cell-laden RGD-functionalized PEG hydrogels (1 × 10^6^ cells per mL) were incubated with QD-peptide complexes (20 nM) in culture medium, and 3D confocal volumes were excited with multiphoton pulsed laser light (740 nm, 16 mW average power at the objective). (c) Confocal images of GFP-expressing HeLa cells entrapped within the 3D hydrogel showing the distinct internalization of QDs without (c–e) and following (f–h) two-photon irradiation (Green: GFP; Blue: DAPI; Red: CdSe/ZnS QDs). GFP brightness is reduced in these images (e) and (h) so that nuclei and QDs are more visible. Scale bars: 10 μm.

We further demonstrated the potential for activating cell–nanomaterial interactions with two-photon excitation, where a high power pulsed laser with a very short pulse was applied in confocal microscopy, providing the potential for deep tissue manipulation. The high photon density in the focused region enhances the probability that a chemical group/molecule absorbs two photons quasi-simultaneously. To this end, we incorporated an *o*-nitrobenzyl ether moiety into the peptide chain between the blocking sequence and cell-penetrating sequence (Fig. 5a and S1†) and attached this peptide to the surface of streptavidin-coated CdSe/ZnS QDs *via* biotin–streptavidin affinity. Compared to the amide bond (Fig. 1a), the ester bond is more sensitive to light irradiation and gives a higher quantum yield of photocleavage. It was previously demonstrated that the irreversible photocleavage of an *o*-nitrobenzyl ether moiety into nitroso- and acid-terminated by-products *via* two-photon irradiation could be used to dynamically control the properties of 3D hydrogels with photocleavable crosslinkers.^43^ Here, we incubated the peptide-modified QDs (20 nM) with HeLa cells encapsulated in the 3D matrix of 8-arm polyethylene glycol (PEG) hydrogels (Fig. 5b). We prepared the hydrogels with 8% w/v 8-arm PEG acrylate, 5 mM RGD peptide (CGGRGDSP), and 6 mM PEG dithiol crosslinker, where the RGD peptides were used to promote cell viability and adhesion to the hydrogel network. To precisely locate viable cells within the 3D hydrogel, HeLa cells expressing green fluorescent protein (GFP) were used. As shown in Fig. 5c–h, two-photon excitation (740 nm) was applied within precisely defined 3-dimensional regions of the hydrogel, resulting in increased intracellular red fluorescence indicating the significant enhancement of QD uptake into HeLa cells.

**Fig. 6:**
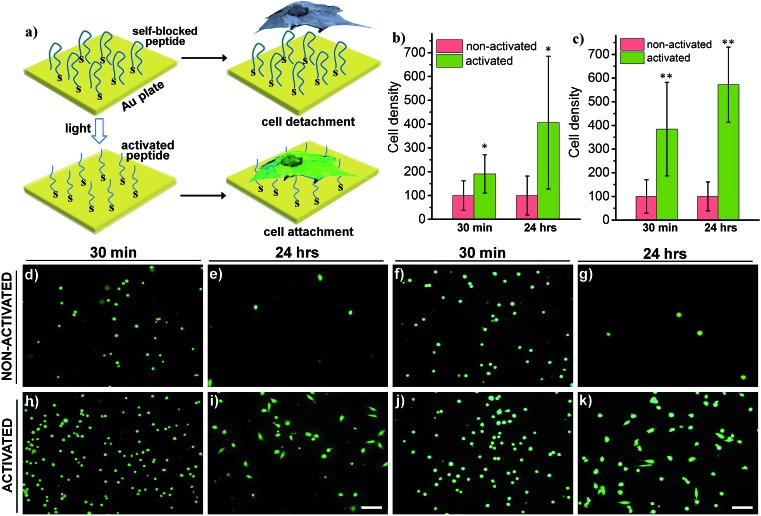
(a) Schematic of a light-activatable Au surface coated with HS-R7E7. The removal of blocking peptides renders the Au surface highly positive, allowing efficient surface binding of cells. (b and c) Cell density on the ligand-modified Au surface with and without light irradiation: (b) MDA cell; (c) HeLa cell. Calcein AM-stained cells were counted 30 min and 24 h after plating. (d–k) Fluorescence images of HeLa (d, e, h, and i) and MDA cells (f, g, j, and k) cultured on the Au surface without (d–g) and with (h–k) light activation. Scale bars: 100 μm. *: *p* < 0.05; **: *p* < 0.01 non-activated *vs.* activated.

The application of CPPs is usually hampered by their poor selectivity towards target cells. We demonstrated enhanced uptake of photo-activated biomaterial-conjugated CPPs, revealing their great potential for controlling delivery of small molecule drugs, biomolecules and nanomaterials. Uptake can be controlled both spatially and temporally, potentially reducing side effects of toxic drugs and increasing the effectiveness of therapies. Moreover, stabilising and neutralising positively charged CPP sequences with anionic peptides until controlled photo-activation occurs could enhance the circulation time in the blood stream. By using precisely controlled and deep tissue penetrating two-photon excitation, we believe that this system will be applicable to *in vivo* studies providing 3D resolution for biomaterial delivery.

Switchable biological surfaces have important applications in cell-based diagnostics and tissue engineering.^44^,^45^ The photo-triggered conversion from a neutral zwitterionic ligand to a highly positive peptide can be exploited to construct a photo-responsive surface with tuneable cell binding capacity. Hydrophilic zwitterionic peptides have been established as effective antifouling agents,^46^ whereas a positive surface (*e.g.*, polylysine) often favours cell attachment. Here, we conjugate HS-R7E7 to an Au surface (**Fig. 6a**), and significant increases in cell attachment to the light-activated surfaces were observed compared to non-activated surfaces (**Fig. 6b–k**). Moreover, cells plated on the photo-activated Au surface could spread and remain attached, as depicted by their spread morphology and the relative increase in cell density after 24 h, yet those on non-activated surfaces were unable to do so, as indicated by their rounded shape and low cell density. Therefore, our strategy can be utilized to controllably switch the surface properties of a material towards supporting cell attachment and growth, which could be used to prepare patterned biological surfaces with photomasks that regulate cell patterning and migration.^44^,^47^,^48^

## Conclusions

In conclusion, we report a general strategy to develop photo-switchable cell–biomaterial interfaces with a new class of photo-labile ligands conjugated onto different biomolecules (*e.g.*, FITC and avidin) and nanomaterials (*e.g.*, quantum dots, polystyrene particles, Au nanostars, and liposomes). We demonstrated enhanced cellular uptake of cargoes by light activation up to 9-fold, as shown for QDs. We also applied our system to demonstrate controllable cancer drug delivery and showed the possibility to engineer smart biointerfaces with tuneable cell attachment/growth properties controlled by light irradiation. The photo-reaction is highly cytocompatible (Fig. S10†), facilitating a wide range of new technologies to regulate cell–material interactions, and control cell attachment, migration and culture in a 3D scaffold, and pave the way towards novel and exciting translatable applications. In particular, incorporating NIR-responsive crosslinkers (Fig. 5)^33^,^43^ and employing spatiotemporally controlled two-photon excitation will address the issues of low penetration depth and the absorption of UV-light in biological tissues.

## Experimental

### Materials

Rink amide resin, Fmoc-protected amino acids, *N*,*N*′-dimethyl formamide (DMF), dichloromethane (DCM), 20% piperidine in DMF, *O*-benzotriazole-*N*,*N*,*N*′,*N*′-tetramethyluronium-hexafluoro-phosphate (HBTU), and diisopropylethylamine (DIEA) were purchased from AGTC Bioproducts. The Fmoc-photolinker 4-{4-[1-(9-Fluorenylmethyloxycarbonylamino)ethyl]-2-methoxy-5-nitrophenoxy}butanoic acid and hydroxyethyl photolinker were obtained from IRIS Biotech GmbH. A Qdot 605 ITK Streptavidin Conjugate Kit was obtained from Life Technologies (U.K.). Carboxylate-modified polystyrene nanoparticles (Latex beads, 20 nm, *λ*_ex_ ∼470 nm, *λ*_em_ ∼505 nm) were obtained from Sigma-Aldrich (U.K.). Lipids were purchased from Avanti Polar Lipids (Alabaster, AL). DSPE-PEG-NHS was purchased from Nanocs. 8-arm PEG (40 000 MW) was purchased from Jenkem (U.S.A.). All the other chemicals were purchased from Sigma-Aldrich (U.K.) and used without further purification.

### Solid phase peptide synthesis (SPPS)

Peptides were synthesized manually using standard fluorenylmethoxycarbonyl (Fmoc) chemistry protocols. The Fmoc protecting group was removed with 20% piperidine/DMF. Fmoc-protected amino acids were activated with 4 molar equivalents of the Fmoc-protected amino acids, 3.95 molar equivalents of HBTU, and 6 molar equivalents of DIEA in DMF. The coupling solution was added to the resin, and the coupling reaction was allowed to proceed for two to three hours. Kaiser tests were performed after each Fmoc deprotection and coupling step to monitor the presence of free amines. Peptides were cleaved in the mixture of trifluoroacetic acid/triisopropylsilane/H_2_O (95 : 2.5 : 2.5) for four hours. The peptides were purified using reversed phase preparative high performance liquid chromatography (HPLC; Shimadzu) in an acetonitrile/water gradient under acidic conditions on a Phenomenex C18 Gemini NX column (5 micron pore size, 110 Å particle size, 150 × 21.2 mm). The purified peptide mass was verified by matrix-assisted laser desorption spectroscopy (MALDI; Waters).

### 8-arm PEG acrylate synthesis

8-arm PEG 40K acrylate was synthesized by reacting hydroxy-terminal 8-arm PEG (MW 40 000) with 12 eq. acryloyl chloride (relative to –OH) and 13 eq. diisopropylethylamine in anhydrous DCM overnight. The pure product was recovered by celite filtration and precipitation in ice cold (–20 °C) diethyl ether. Acrylate functionalization (83%) was confirmed by NMR.

### Light irradiation

The light-irradiation experiments were conducted using a UV lamp (UVLMS-38 EL Series 3 UV™, 365 nm, 1.3 mW cm^–2^) with a distance of 10 cm between the sample and the lamp. To test the light-responsiveness of peptides, 0.1 mM peptide was dissolved in phosphate buffer (10 mM, pH 7.0). The solution was exposed to UV light and the UV-vis spectra were recorded every 30 seconds.

### Synthesis of Au nanostars

Gold nanostars were prepared using a seeded, HEPES/hydroxylamine reduction approach similar to that reported by Maiorano *et al.* with minor adjustments. Briefly, a solution, consisting of 38.5 mL Milli-Q water, 18.75 mL of 100 mM HEPES buffer at pH 9.6, 750 μL of fresh 40 mM hydroxylamine and 750 μL of the as-prepared citrate gold nanoparticle seeds, was prepared in a 100 mL conical flask. A sufficiently large magnetic stirrer bar was used to achieve effective mixing at a stirring rate of 1450 rpm. To this rapidly mixing solution, 22.5 mL of 1 mM HAuCl_4_·3H_2_O was added at a rate of 2 drops per second. Upon complete addition of the HAuCl_4_·3H_2_O, the stirring speed was reduced to 400 rpm, and the stirring was continued for a further 15 minutes, and subsequently, 80 μL of 1 wt% aqueous solution of Tween-20 surfactant was added. The particles were centrifuged at 4000 rpm for 30 minutes for 3 cycles including brief sonication to assist resuspension such that the final concentration of Tween-20 was approximately 0.0001 wt%. The particles were then passed through a 0.2 μm PES filter membrane. The peak maximum of the longitudinal plasmon absorption was at 820 nm, and the final concentration of gold [Au] = 2.6 mM.

### Synthesis of 13 nm citrate-capped gold nanoparticle seeds

180 mL of Milli-Q water in a 250 mL round-bottom flask (2-neck) was brought to reflux in an oil bath and stirred gently. A solution of 79 mg of HAuCl_4_·3H_2_O in 5 mL of Milli-Q water was then added, and the stirring speed was increased as high as possible. Within 5 minutes, a 10 mL solution of 2 wt% trisodium citrate dihydrate in Milli-Q water (pre-heated to 70 °C) was injected rapidly. Reflux was continued for a further 5 minutes with fast stirring before being removed from the oil bath and allowed to cool to room temperature. The particles were then allowed to cool and stored at 4 °C prior to use.

### Surface functionalization of Au nanostars

To functionalize Au nanostars, 10 μL of 1.0 mM HS-PEG2K and 50 μL of 0.5 mM HS-R7E7 were added to 2 mL Au nanostar solution (5.5 mM). The mixture was incubated at room temperature overnight. After centrifugation at 10 000 rpm for 10 minutes, the supernatant was discarded, and the pellet was re-dispersed in phosphate buffer.

### Surface functionalization of quantum dots (QDs)

Peptide conjugation on CdTe quantum dots was achieved *via* biotin–streptavidin chemistry. Briefly, 10 μL of commercial streptavidin-coated CdTe QDs (4 μM) was incubated with 1.0 mL 10 μM biotinylated photo-cleavable peptide (biotin-R7E7) for 1.0 hour. The solution was centrifuged at 32 490 rpm for 2 hours with an ultracentrifuge to remove the excess unbound peptides. The supernatant was discarded, and the QDs were re-dispersed in phosphate buffer.

### Surface modification of fluorescent polystyrene (PS) nanoparticles

Typically, 80 μL of carboxylic acid-modified polystyrene particles (20 nm, 25 mg mL^–1^) were dispersed in 2 mL MES buffer (pH 5.7, 20 mM). After that, 20 mg of EDC and 20 mg of NHS were added to activate the carboxyl group. The mixture was incubated for 2 hours at room temperature before the addition of NH_2_-R7E7 (2 mg in 50 mM phosphate buffer, pH 8.0). After incubation overnight, PS particles were centrifuged, and the suspension was discarded to remove excess chemical reagents. The PS particles were washed with PBS again before the cell experiments.

### Preparation of ligand-functionalized liposomes

The liposome sample was prepared by the extrusion method. In detail, 1.5 mg of 1,2-dioleoyl-*sn*-glycero-3-phosphoethanolamine-*N*-[4-(*p*-maleimidophenyl)butyramide] (sodium salt) and 2 mg of HS-R7E7 were incubated in methanol overnight. To this mixture, 2.5 mg of 1-palmitoyl-2-oleoyl-*sn*-glycero-3-phosphocholine (POPC) and 0.1 mg of Texas Red® 1,2-dihexadecanoyl-*sn*-glycero-3-phosphoethanolamine, triethylammonium salt (Texas Red® DHPE) in chloroform were added. The solution was dried under a N_2_ flow. 0.5 mL of PBS buffer was added, and the mixture was vortexed for 30 seconds. The lipid suspension was extruded through a 200 nm polycarbonate membrane 31 times. The unreacted peptide was removed by passing through a Sephadex G100 column.

### Surface modification of Au plate

The Au surface was rinsed with ethanol three times and dried in air. After that, 1 mg mL^–1^ of HS-R7E7 solution was applied to the Au surface and incubated for 5 hours. The Au surface was then washed with water and ethanol several times. The Au surface was further incubated with a HS-PEG1000 solution (1 mM) to block the surface.

### Characterization

UV-Vis spectra were recorded with a Lambda 25 spectrometer (Perkin Elmer). Fluorescence spectra were recorded with a Fluorolog fluorometer (Horiba). The fluorescence measurements of cell experiments were conducted with a SpectraMax M5 plate reader.

### Cell culture

All cell culture reagents were from Thermo Fisher Scientific (Loughborough, UK) unless otherwise stated. MDA-MB-231 cells were purchased from ATCC (Teddington, UK), HeLa cells from DSMZ (Braunschweig, Germany), and GFP-HeLa cells from Cell Biolabs, Inc. (San Diego, CA, USA). Cell lines were cultured under standard conditions (37 °C and 5% CO_2_) in DMEM supplemented with 10 v/v% fetal bovine serum and 1 v/v% penicillin streptomycin and split using trypsin–EDTA upon confluence. For uptake experiments (FITC, avidin, QDs, PS, Au nanostars, liposomes and camptothecin-loaded PLGA nanoparticles), cells were plated in 96 well plates at 10 000 cells per well and left to attach overnight. The next day, the medium was replaced by the corresponding constructs diluted in PBS : medium (1 : 1) and again incubated overnight. After 24 hours, the medium was discarded and cells were gently washed with PBS 3 times before fluorescence intensity was measured or the alamarBlue® test was performed (PLGA nanoparticles).

For live activation of QD-R7E7 complexes, MDA-MB-231 cells were incubated with 20 nM QDs and irradiated for 0, 5 and 15 minutes. 24 hours later, epifluorescence images were taken, and cells were trypsinized and analyzed by FACS.

For Au attachment experiments, cells were detached using 0.05 w/v% trypsin–EDTA, counted and plated at 100 000 cells per mL in DMEM without supplements to avoid interference from the charged molecules in the serum. 30 minutes after plating, the medium was removed and the samples were washed with PBS. Cell attachment was evaluated after staining with calcein AM, and 4–6 pictures per sample were taken randomly. The substrates were fed with complete medium and incubated overnight. After 24 hours, the samples were again stained with calcein AM, and the same number of images was acquired.

Viability of the cultures after incubation with the different nanomaterials was evaluated using either LIVE/DEAD staining or alamarBlue®, both following the manufacturer's instructions.

### Fluorescence-activated cell sorting (FACS)

For FACS experiments, cells were plated in a 12 well plate at an equivalent density to the uptake experiments and left to attach overnight. The next day, they were incubated with the different compounds or nanomaterials for 24 hours and detached with 0.05 w/v% trypsin–EDTA. Non-treated cells were used as a control. Fluorescence was measured using a Fortessa cytometer (BD, Oxford, UK).

### Cell encapsulation in PEG hydrogels

15 mm diameter glass coverslips were thiol-functionalized with 3-mercaptopropyl trimethoxysilane in acetone for 10 minutes, rinsed in acetone, heated at 80 °C for 10 minutes, cooled and stored at –20 °C. Cell-laden hydrogels were prepared with 8% w/v 8-arm PEG acrylate, 5 mM RGD peptide (CGGRGDSP), 6 mM PEG dithiol crosslinker (MW 1000), and 1 × 10^6^ cells per mL in DMEM with 20 mM HEPES, pipetted into silicone moulds with 6 mm diameter and 500 μm thickness, on top of thiol-functionalized glass coverslips, and covered with Rain-x treated glass coverslips. After 12 minutes, gelation was complete, and individual gels attached to 15 mm coverslips were transferred to 24-well plates with the cell culture medium and cultured for 2 days before 2-photon photoactivation experiments.

### Two-photon photoactivation

Cell-laden hydrogels were incubated with 20 nM QD-peptide in the culture medium for 3 hours, and several 3-dimensional regions of interest at the center of the cell-laden gels (300 × 300 μm *x–y*, 200 μm in *z* with 5 μm *z*-spacing) were selectively exposed to multiphoton pulsed laser light (740 nm, 40 × 0.8 NA water immersion objective, 16 mW average power at the objective) to photo cleave the QD-bound peptide, using an upright multiphoton confocal microscope (Scientifica, Uckfield, UK). Hydrogels were incubated overnight to allow for cellular uptake of the photoactivated QD-peptide.

### Microscopy

For conventional fluorescence microscopy, cells were imaged live using an IX51 epifluorescence inverted microscope (Olympus, Southend-on-Sea, UK). For confocal microscopy, cells were plated on glass bottom microslide chambers (Ibidi, Glasgow, UK) and treated as above. All washes were performed with PBS. After incubation, cells or cell-laden hydrogels were washed and fixed for 15 minutes in 4 w/v% paraformaldehyde, washed again in PBS and stained with WGA (where indicated, conjugated to 488-AlexaFluor for FITC-R7E7 and PS-R7E7 or 594-AlexaFluor for QD-biotin-R7E7) for 15 minutes at room temperature. Samples were then washed and incubated with DAPI for 5 minutes, washed and mounted with a Vectashield (Vector Laboratories, Peterborough, UK). Cell-laden hydrogels were mounted with a FluorSave (Calbiochem USA). Imaging was performed using a SP5 MP/FLIM inverted confocal microscope (Leica Microsystems, Milton Keynes, UK). For hydrogel experiments, GFP-positive cellular uptake of QDs was compared at the gel center (photoactivation region) *versus* gel edge (receiving no activation).

### Statistics

Results are presented as mean ± standard deviation unless specified otherwise. Statistical analysis was performed using SPSS 22 and Prism software. Distributions were assumed normal, and differences were analyzed using the Student's *t*-test. Differences were considered statistically significant when *p* < 0.05 (*) and very significant when *p* < 0.01 (**).

## Conflicts of interest

There are no conflicts to declare.

## Supplementary Material

Supplementary information
