# Supplementary material for: Activatable cell–biomaterial interfacing with photo-caged peptides
Source: Chem Sci. 2018 Nov 16;10(4):1158–67. doi: 10.1039/c8sc04725a (PMC6349021; doi:10.1039/c8sc04725a)
Supplement: Supplementary file 1 [file SC-010-C8SC04725A-s001.pdf]

Electronic Supplementary Material (ESI) for Chemical Science.
This journal is © The Royal Society of Chemistry 2018

Supporting Information

Activatable cell-biomaterial interfacing with photo-caged peptides

Yiyang Lin,[a]† Manuel M. Mazo,[a]† Stacey C. Skaalure,[a] Michael R. Thomas,[a] Simon R. Schultz,[b] Molly M. Stevens*[a]

[a] Department of Materials, Department of Bioengineering and Institute for Biomedical Engineering, Imperial College London, Exhibition Road, London SW7 2AZ, UK.

[b] Department of Bioengineering and Centre for Neurotechnology, Imperial College London, London SW7 2AZ, UK

E-mail: m.stevens@imperial.ac.uk

[+] These authors contributed equally to this work.

(a)

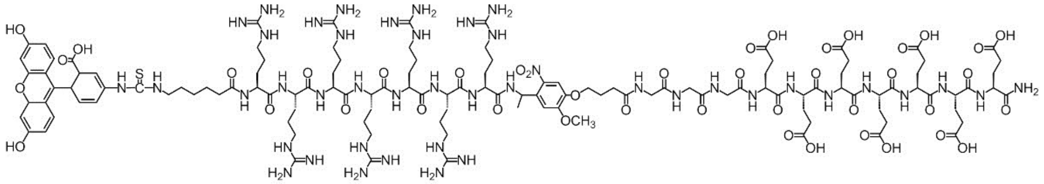

(b)

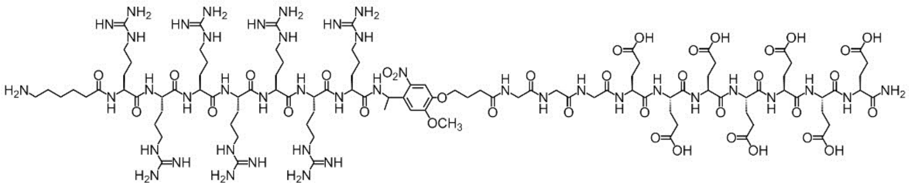

(c)

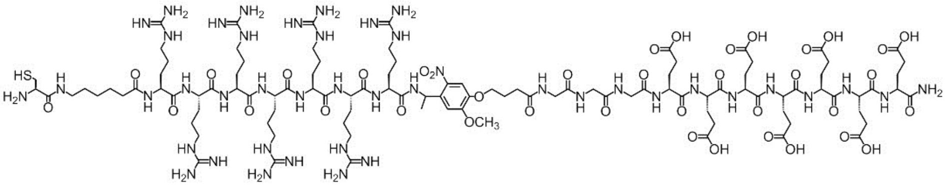

(d)

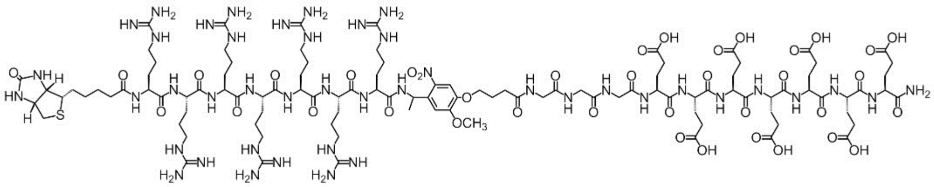

(e)

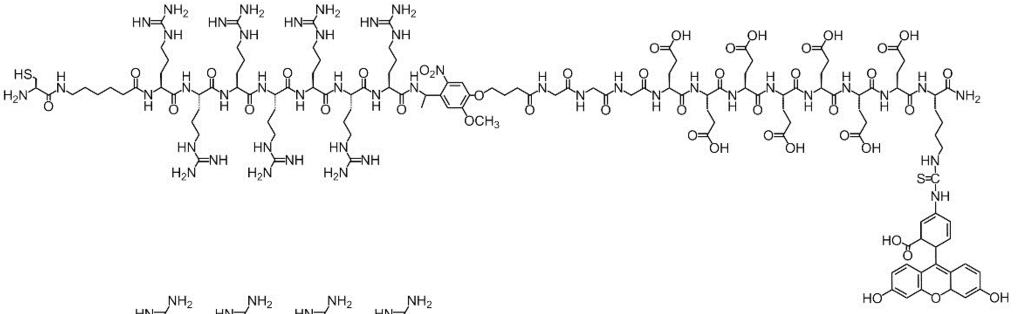

(f)

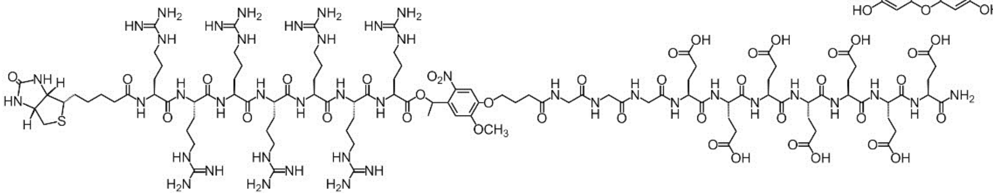

Figure S1. Molecular structures of light-activatable ligands with different functional groups at the N-terminus. (a) FITC-R7E7; (b) H_2N-R7E7; (c) HS-R7E7; (d) Biotin-R7E7; (e) HS-R7E7-FITC; (f) Biotin-R7E7 (two-photon cleavable). The peptides contain a hepta-arginine as the cell-penetrating sequence, a hepta-glutamic acid as the CPP-blocking group, a nitrophenyl group as the photo-cleavable linkage, and different functional groups (i.e., biotin, amine, thiol, and FITC) at the N-termini.

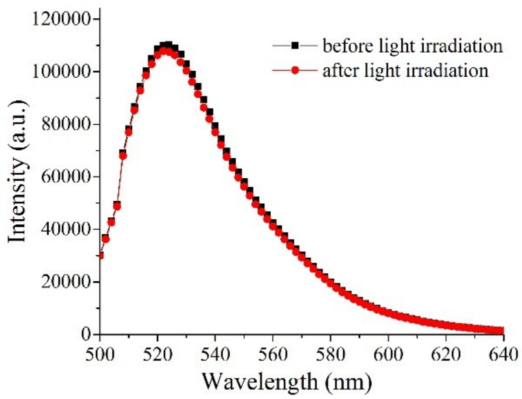

Figure S2. Fluorescence spectra of FITC-R7E7 (50 µM in PBS) before and after 365 nm light irradiation for 10 minutes. No significant changes of fluorescence emission were observed.

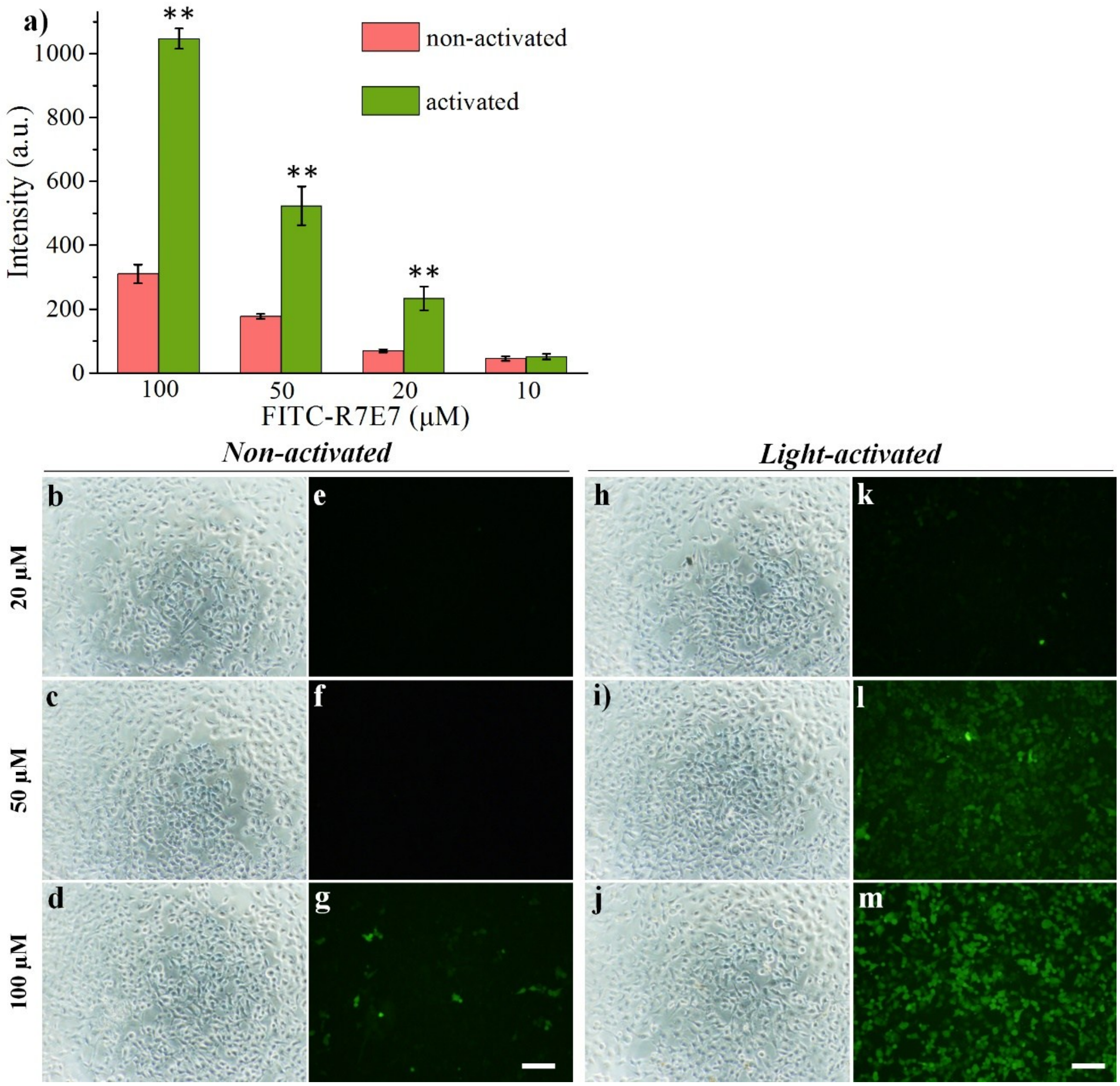

Figure S3. (a) Fluorescence intensity of HeLa cells after peptide internalization. The cells were incubated with FITC-R7E7 at 100, 50, 20 and 10 μM for 24 hours before the measurement. (b-d) Optical microscopy and (e-g) fluorescence microscopy images of HeLa cells incubating with FITC-R7E7 without 365 nm light irradiation. (h-j) Optical and (k-m) fluorescence microscopy images of HeLa cells incubating with FITC-R7E7 with 365 nm light irradiation. Scale bar: 100 μm. **: $p < 0.01$ non-activated vs light activated.

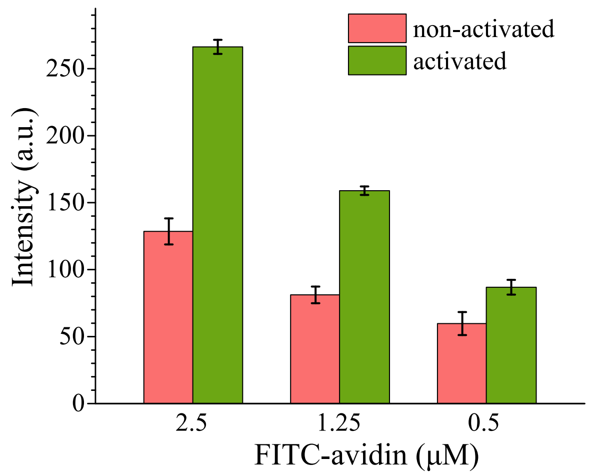

Figure S4. Cellular uptake of FITC-avidin by MDA-MB-231 cells. FITC-avidin solution was incubated with biotin-R7E7 for 30 minutes to allow complete avidin-biotin binding. The mixture was incubated with cells for 24 hours before measurement of fluorescence. *: $p < 0.05$; **: $p < 0.01$ non-activated vs light activated.

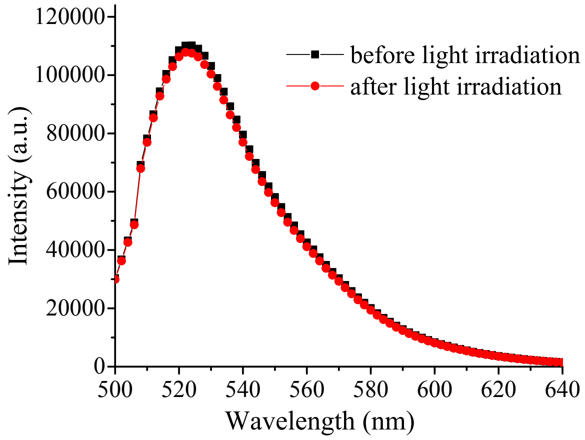

Figure S5. Fluorescence spectra of QD-R7E7 (20 nM in PBS) before and after 365 nm light irradiation for 10 minutes. No significant changes in fluorescence intensity were observed.

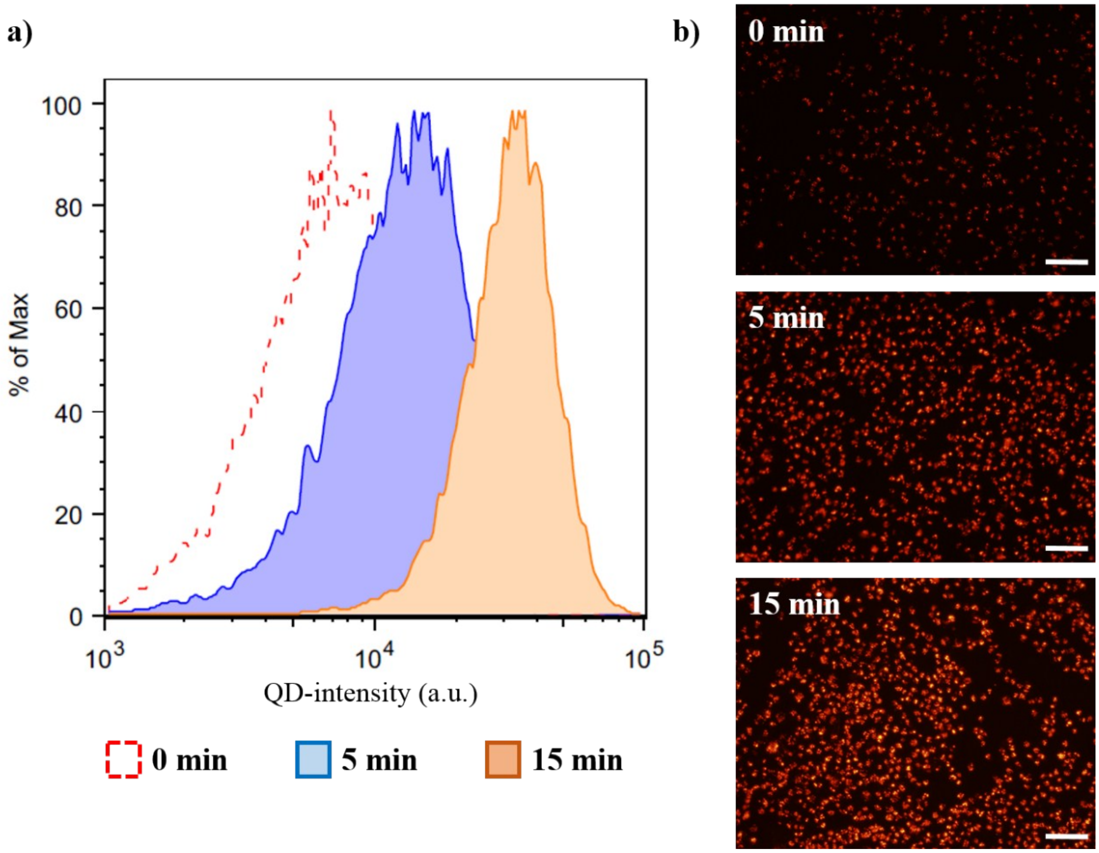

Figure S6. *In situ* photo-activated QD entry into MDA-MB-231 cells. (a) FACS analysis and (b) epifluorescence images show an increased cellular uptake of QDs with irradiation time (0, 5 and 15 minutes). MDA-MB-231 cells were incubated with photo-activated peptide modified QDs (20 nM) and irradiated by 365 nm UV light for 0, 5 and 15 min. After incubating for 24 hrs, epifluorescence images were taken, and the cells were analyzed by FACS. Scale bars: 100 μm.

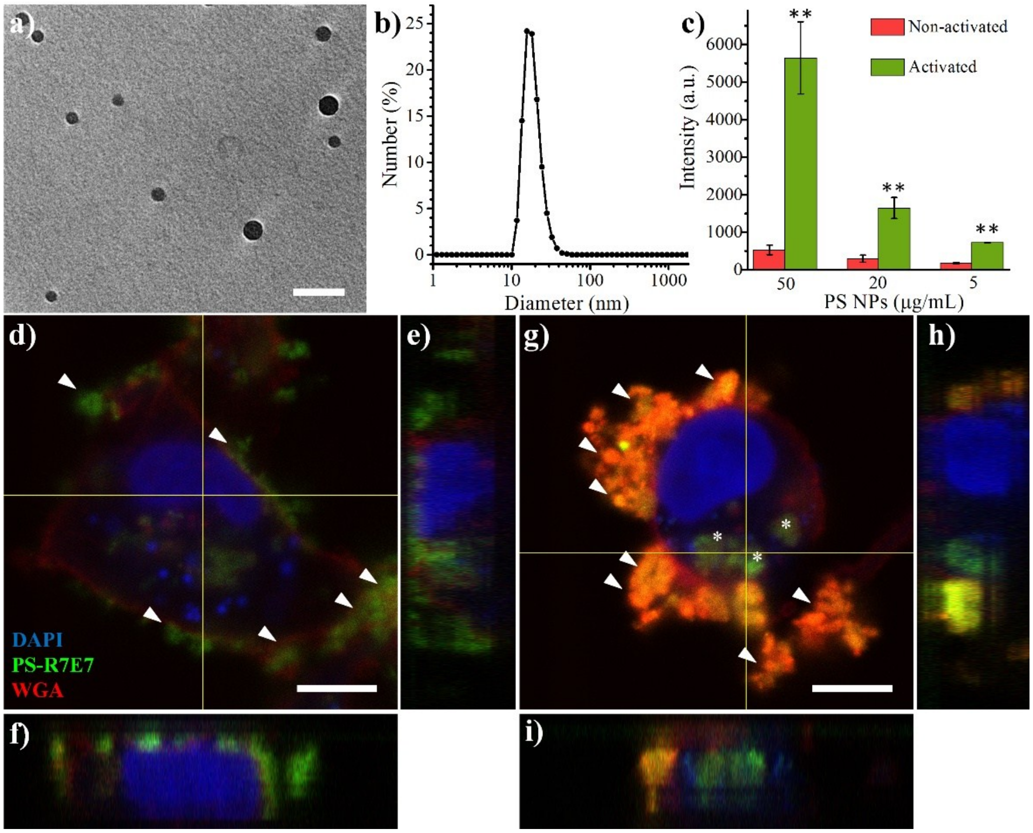

Figure S7. (a) Transmission electron microscopy image and (b) dynamic light scattering of peptide functionalized polystyrene (PS) particles (20 nm). Scale bar: 100 nm. (c) Fluorescence intensity of MDA-MB-231 cells after incubating with different amounts of polystyrene particles. Non-internalized particles were removed by washing each well three times. Confocal images of MDA-MB-231 cells incubated with NH_2-R7E7 conjugated PS: (d) PS particles coupled to the non-activated peptide show mainly pericellular location after 24 hours (d, arrowheads), whereas the activated ones have either been incorporated into the cytoplasm (g, asterisks) or are engulfed by the cell membrane (g, arrowheads). Scale bar: 20 μm. **: $p < 0.01$ non-activated vs light activated.

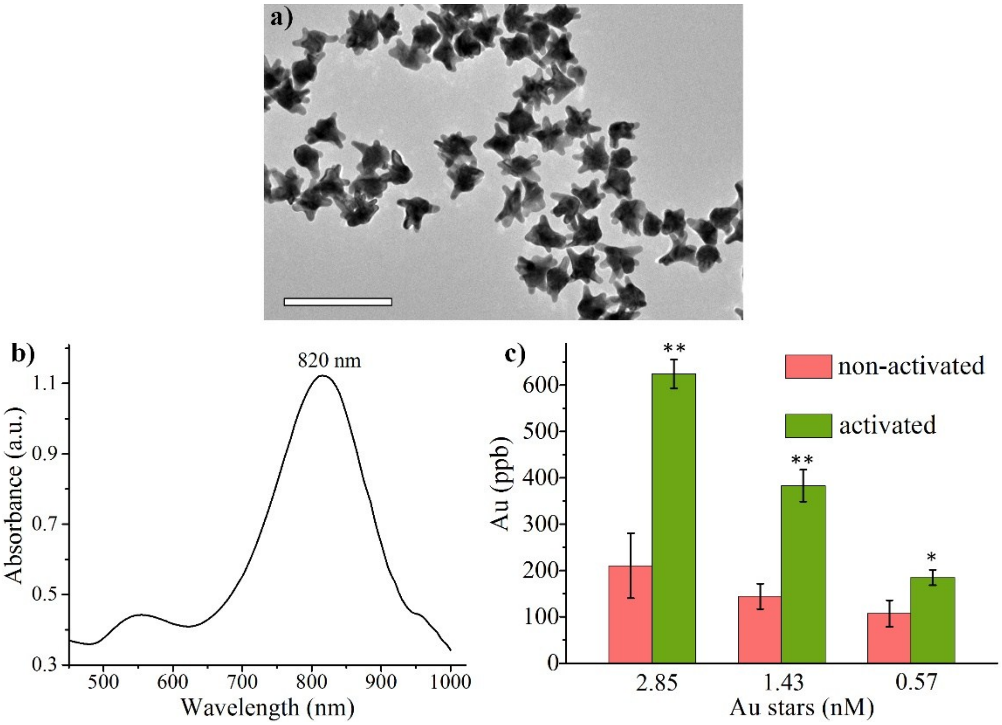

Figure S8. (a) TEM images of Au nanostars. Scale bar: 200 nm. (b) LSPR spectrum of Au nanostars; (c) ICP-MASS of Au element from MDA-MB-231 cells after incubating with peptide-functionalized Au nanostars for 24 hours. The Au nanostars are functionalized with HS-R7E7 peptides via Au-thiol bond. *: $p < 0.05$; **: $p < 0.01$ non-activated vs light activated.

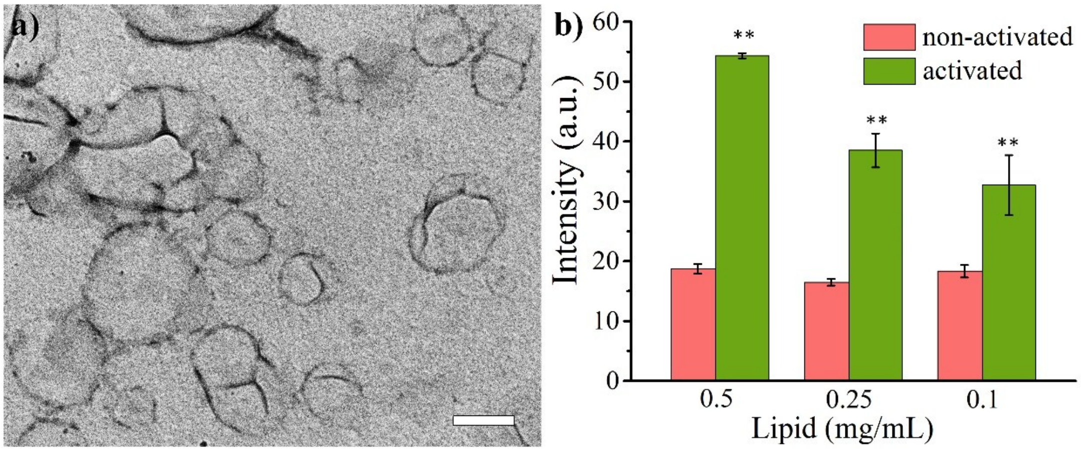

Figure S9. (a) TEM images of liposomes; (b) Fluorescence intensity of MDA-MB-231 cells after incubating with fluorophore-doped photo-liable peptide conjugated liposomes for 24 hours. **: $p < 0.01$ non-activated vs light activated. Scale bar: 200 nm

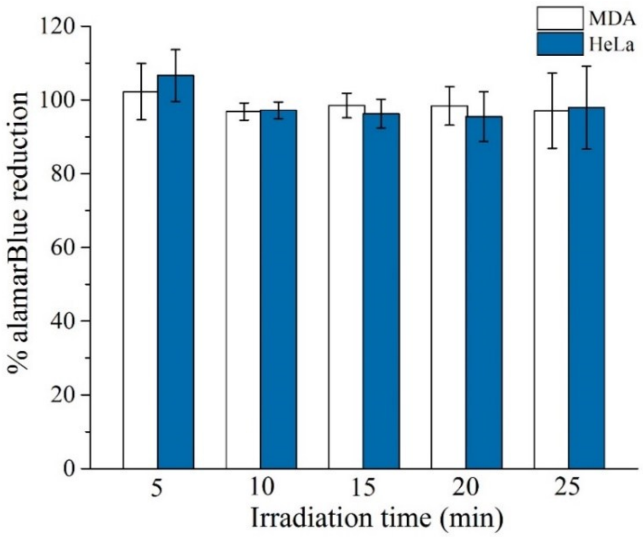

Figure S10. Cellular toxicity of MDA-MB-231 and HeLa cells after UV light irradiation for 5, 10, 15, 20 and 25 minutes.
